# Not “woke” agendas, basic human rights: reimagining disaster preparedness through Maslow’s lens

**DOI:** 10.3389/fpubh.2025.1533117

**Published:** 2025-03-06

**Authors:** Omolola E. Adepoju

**Affiliations:** Department of Health Systems and Population Health Sciences, Tilman J. Fertitta Family College of Medicine, University of Houston, Houston, TX, United States

**Keywords:** disaster & psychological consequences & risks & interventions & prevention, disaster, underserved, policy, Maslow Hierarchy, community engagement

## Abstract

Disaster preparedness has become increasingly essential in mitigating the personal impacts of climate change. However, vulnerable communities struggling with preparedness continue to face disproportionate risks and outcomes, particularly in Houston—a city affected by successive, historic federally declared disasters. This piece seeks to reframe disaster preparedness, response, and recovery through the lens of Maslow’s Hierarchy of Needs, arguing that preparedness should be viewed as a fundamental survival need. Just as food, water, and shelter are critical for human survival, effective disaster preparedness ensures the ability to respond and recover during crises. For communities already bearing the compounded burdens of climate change and successive disasters, preparedness is not merely a response but a prerequisite for resilience. In light of this, this article advocates for policies and interventions that prioritize accessible, community-centered preparedness strategies.

Given the increasing frequency of natural disasters (1) precipitated by climate change—the importance of bottom-up community-driven efforts—led by residents of communities affected by these disasters—is paramount to better understanding and addressing the impacts of successive disaster events on environmental health disparities. This is the reality in Houston, which has endured 33 federally declared disasters since 2000 (2), including Hurricane Harvey, which caused an estimated $125 billion in damages and disproportionately impacted low-income and minority communities (3). Compared to other disaster-prone cities, Houston’s response and recovery efforts have faced significant delays. For instance, nearly two years after Hurricane Harvey, over 15% of affected households were still waiting for housing assistance (4). These disparities underscore the need for localized, community-driven strategies that prioritize the most vulnerable populations in disaster preparedness and response efforts.

As part of an academic-community effort focused on assessing the impact of successive disaster events on mental health and social needs, we held three (3) town-hall style community conversations examining housing insecurity and its role in disaster vulnerability. Facilitated by a team from the University of Houston and community organizers, these events brought together residents of three historically Black Houston communities—Kashmere Gardens, Greater Third Ward and Fifth Ward. The conversations focused on realities faced during Houston’s successive disaster events, emphasizing their priorities and needs in the recovery process. Conversations explored how pre-existing housing inequalities make marginalized communities more vulnerable to disasters, including how substandard housing, gentrification, lack of access to insurance, and inadequate disaster preparedness resources, impede preparedness and response. Conversations also included policy interventions to reduce these vulnerabilities and promote more equitable disaster response and recovery efforts.

Initial discussions framed climate as a concern for the elite, but as conversations progressed, it became evident that while climate-induced disasters affect everyone, their consequences disproportionately impact underserved communities. For example, community residents repeatedly reported being the first to lose power and the last to have power restored. Handymen and service workers were also more likely to prioritize more affluent neighborhoods, leaving underserved areas with prolonged disruptions and inadequate responses during crises. As the conversation progressed, one community member, Billy, blurted out that disaster preparedness and response in these communities are not a “woke agenda, they are basic human rights.”

As I drove home, I thought of Billy’s comment using Maslow’s Hierarchy of Needs and how spot-on he was without even realizing it. Maslow’s “Hierarchy of Needs” pyramid is a psychological theory that suggests that human beings require certain fundamental needs in order to function and progress toward higher levels of well-being and self-actualization (5). The base of the pyramid reflects the most essential needs, like food, water, and shelter, followed by safety and security. Once these foundational needs are met, individuals can move on to higher levels, including love and belonging, esteem, and finally, self-actualization. Disaster preparedness aligns closely with the safety level of this hierarchy, as it encompasses the strategies and resources necessary to protect individuals and communities to ensure they are safe. Similarly, FEMA’s preparedness model underscores the importance of mitigation, preparedness, response, and recovery as interconnected phases that ensure resilience before, during, and after disasters (6). By integrating both models, we emphasize that preparedness is not just an individual responsibility but a structural necessity—one that requires proactive planning, equitable resource distribution, and community-driven efforts to safeguard fundamental human needs.

For underserved communities, where social determinants such as poverty, lack of access to healthcare, and inadequate housing compound vulnerability, prioritizing disaster preparedness is crucial. Climate change and natural disasters cannot be viewed as a secondary concern, as they directly threaten the fundamental needs of these marginalized communities. Without proper housing, adequate resources, and support systems in place to prepare for and recover from these disasters, individuals in these communities cannot build the secure environment necessary to thrive or move toward higher levels of well-being. By viewing climate change through this lens, we can address not only the immediate safety needs of these populations but also foster resilience and empowerment, ensuring that our underserved communities have the resources and support needed to navigate and adapt to environmental changes effectively. This approach not only emphasizes the intersection of climate and health but also reinforces the idea that equitable access to climate resilience is a basic human right, ensuring that every individual, regardless of their socioeconomic status, has the opportunity to live safely and healthily in the face of environmental challenges.

Social vulnerabilities increase the risk of disasters for marginalized communities. Individuals residing in communities with higher social vulnerabilities often have fewer resources to prepare for, respond to, and recover from disasters, which leaves them more exposed to harm. Among U.S.’ largest counties, Harris County has the highest vulnerability score at 0.72, meaning it is more susceptible to disaster impacts than 72% of U.S. counties (7, 8). In contrast, Fort Bend County scores 0.29, and Montgomery County scores 0.39, indicating they are less vulnerable to such effects. Substandard housing, gentrification, lack of access to insurance, and inadequate disaster preparedness resources further hinder effective preparedness and response in underserved communities. While climate-induced disasters affect everyone, their consequences are not felt equally.

Consequently, there is an urgent need for actionable steps from policymakers, local governments, and community organizations to address disaster-proofing and equitable resource allocation. Policymakers and local governments can prioritize the development of resilient infrastructure by enacting and enforcing robust building codes, while embedding disaster preparedness into urban planning frameworks. Furthermore, they must ensure that marginalized communities receive equitable access to resources and support, fostering collaborative partnerships among stakeholders to implement tailored, context-specific interventions that address the unique needs of diverse populations. Community organizations can play a pivotal role by mobilizing resources, raising awareness, and providing direct support to vulnerable communities, ensuring that preparedness efforts are inclusive and locally informed.

Critics may argue that prioritizing disaster-proofing and equitable resource allocation could stretch Houston’s budget thin or that underserved communities aren’t the only ones in need of resources. These concerns are valid, however failing to prioritize disaster-proofing and equitable resource allocation now will ultimately cost Houston more in the long run, both financially and in human suffering. Houston’s most vulnerable communities are already bearing the brunt of these disasters, and the impacts will only grow. Investing in disaster resilience for underserved communities is a smart, long-term strategy that can reduce strain on the entire city’s recovery process, saving taxpayer money. With global climate projections indicating that extreme weather events will only become more frequent and severe, the likelihood of complex, multi-hazard events—like the unprecedented Texas Winter Storm—remains high. Billy and the other residents at the town hall understand this harsh reality all too well. Yet, too few leaders have truly listened to their voices. Houston’s community members are not interested in debating climate change—they feel its impact on their homes, their families, and their children every day. They want to know: who will listen? And more importantly, who will act?

While Maslow’s hierarchy provides a valuable lens for conceptualizing disaster preparedness as a fundamental survival need, we recognize its limitations in this context. Specifically, its linear structure fails to account for the complex, interdependent social and systemic factors that shape resilience, and disaster preparedness may not always follow a strict hierarchical order, as Maslow suggests. However, we cannot ignore the real pain that low-income communities face—loss of homes, livelihoods, and lives. By neglecting these needs, we risk further entrenching inequality and widening the gap between those who can recover and those who cannot. The real question is not whether we can afford to prioritize these needs but whether we can afford not to.

## Data Availability

The original contributions presented in the study are included in the article/supplementary material; further inquiries can be directed to the corresponding author.
